# Assessing fracture risk in early stage breast cancer patients treated with aromatase-inhibitors: An enhanced screening approach incorporating trabecular bone score

**DOI:** 10.1016/j.jbo.2016.10.004

**Published:** 2016-10-18

**Authors:** Veronica Mariotti, David B. Page, Oksana Davydov, Didier Hans, Clifford A. Hudis, Sujata Patil, Siddharth Kunte, Monica Girotra, Azeez Farooki, Monica N. Fornier

**Affiliations:** a Rutgers New Jersey Medical School, Department of Internal Medicine, Newark, NJ, United States; b Providence Portland Medical Center/Robert W. Franz Cancer Research Center, Portland, OR, United States; c Mount Sinai St. Luke’s - Roosevelt Hospital, Division of Endocrinology, New York, NY, United States; d Bone and Joint Department, Center of Bone Diseases, Lausanne University Hospital, Lausanne, Switzerland; e Memorial Sloan Kettering Cancer Center, Breast Medicine Service, Weil Cornell Medical College, New York, NY, United States; f Memorial Sloan Kettering Cancer Center, Department of Epidemiology and Biostatistics, Weil Cornell Medical College, New York, NY, United States; g Icahn School of Medicine at Mt Sinai St Luke’s Roosevelt, Department of Internal Medicine, New York, NY, United States; h Memorial Sloan Kettering Cancer Center, Department of Endocrinology, Weil Cornell Medical College, New York, NY, United States

**Keywords:** AIs, Aromatase-Inhibitors, DXA, Dual-energy X-ray absobimetry, FRAX®, Fracture risk assessment tool, TBS, Trabecular bone score, BMD, Bone mineral density, WHO, World Health Organization, EFR, Elevated Fracture Risk, Trabecular bone score, Breast cancer, Aromatase inhibitor, Osteoporosis, TBS, FRAX®, Fracture risk assessment tool, Osteopenia, Manitoba study, Adjuvant

## Abstract

**Introduction:**

Aromatase-inhibitors (AIs) are commonly used for treatment of patients with hormone-receptor positive breast carcinoma, and are known to induce bone density loss and increase the risk of fractures. The current standard-of-care screening tool for fracture risk is bone mineral density (BMD) by dual-energy X-ray absorptiometry (DXA). The fracture risk assessment tool (FRAX®) may be used in conjunction with BMD to identify additional osteopenic patients at risk of fracture who may benefit from a bone-modifying agent (BMA). The trabecular bone score (TBS), a novel method of measuring bone microarchitecture by DXA, has been shown to be an independent indicator of increased fracture risk. We report how the addition of TBS and FRAX®, respectively, to BMD contribute to identification of elevated fracture risk (EFR) in postmenopausal breast cancer patients treated with AIs.

**Methods:**

100 patients with early stage hormone-positive breast cancer treated with AIs, no prior BMAs, and with serial DXAs were identified. BMD and TBS were measured from DXA images before and following initiation of AIs, and FRAX® scores were calculated from review of clinical records. EFR was defined as either: BMD ≤−2.5 or BMD between −2.5 and −1 plus either increased risk by FRAX® or degraded microstructure by TBS.

**Results:**

At baseline, BMD alone identified 4% of patients with EFR. The addition of FRAX® increased detection to 13%, whereas the combination of BMD, FRAX® and TBS identified 20% of patients with EFR. Following AIs, changes in TBS were independent of changes in BMD. On follow-up DXA, BMD alone detected an additional 1 patient at EFR (1%), whereas BMD+ FRAX® identified 3 additional patients (3%), and BMD+FRAX®+TBS identified 7 additional patients (7%).

**Conclusions:**

The combination of FRAX®, TBS, and BMD maximized the identification of patients with EFR. TBS is a novel assessment that enhances the detection of patients who may benefit from BMAs.

## 1 Introduction

Aromatase-Inhibitors (AIs) are commonly used in the treatment of post-menopausal women with a history of hormone receptor-positive breast carcinoma, and have been shown to decrease bone mineral density (BMD) and increase the risk of bone fragility fractures [1].

The National Comprehensive Cancer Network Task Force (NCCN) currently recommends screening of fracture risk in all patients initiating AIs by obtaining clinical history, dual-energy X-ray absorptiometry (DXA) scans and with the use of the fracture risk assessment tool (FRAX®) calculator. If T-score is less than or equal to −2.0 at any site or if the FRAX® 10-year absolute risk of fracture is greater than 20% for any major fracture or greater than 3% for hip fracture, bone modifying-agents (BMAs) such as bisphosphonates or denosumab, are recommended. For women with increased risk of fractures initiating AI therapy, BMAs such as bisphosphonates or denosumab can be recommended, both which have been shown to decrease the risk of bone fracture in the setting of AI therapy [2], [3]. The current gold standard screening tool for the diagnosis of osteoporosis in the absence of fragility fractures is DXA.

Many patients without osteoporotic BMD suffer fragility fractures. It is important to highlight that the majority of fractures actually occur in patients with a T-score above the osteoporotic range [4], making the osteoporosis threshold (BMD T score <2.5) inadequate to identify all patients at risk. Furthermore, BMD does not evaluate the degree of bone microarchitectural deterioration, which may represents an independent factor contributing to increased bone fragility [5].

The trabecular bone score (TBS) is an innovative gray-level texture measurement that utilizes lumbar spine DXA images to discriminate changes in bone microarchitecture [6]. Specifically, TBS measures tridimensional bone areas with different trabecular and microstructural characteristics. TBS has been shown to be an independent indicator of increased fracture risk [7]. Furthermore, the combination of TBS microstructure evaluation with BMD measured by DXA has been shown to be superior to either measurement alone in the assessment of fracture risk [8].

In an effort to optimize the identification of postmenopausal women treated with adjuvant AIs at risk of bone fragility fractures, we evaluated a screening model that integrates the novel TBS tool with FRAX® and DXA. We then studied if our tools represent independent variables in this clinical context, and enumerated the relative contribution of adding TBS to the standard screening approaches most commonly observed in the clinic (BMD±FRAX®).

## 2 Materials and methods

### 2.1 Patient selection

Patients were identified via institutional databases at Memorial Sloan Kettering Cancer Center under an Institutional Review Board waiver of consent. Using DataLine services we identified 309 unique patients who were diagnosed with breast cancer at MSKCC between the years of 2005 and 2012, who were post-menopausal (defined as ≥60 years old or ≥50 years old with amenorrhea for >12 months), were treated with an AI, and who had at least 2 DXAs performed at MSKCC. Through a chart review, we then eliminated patients who were treated with BMAs prior to baseline or follow-up DXA. We also excluded all patients with a BMI over 37, as TBS has not been validated in this population. We then selected the patients who had a baseline DXA within 3 months of starting the AI, and a follow up DXA more than 6 months but less than 36 months after the first one. This search yielded to 100 unique patients who were included in our analysis.

### 2.2 BMD, TBS, and FRAX assessment

As per standard-of-care at MSKCC, BMD from femoral neck, total hip and lumbar spine was measured by DXA (GE-lunar). TBS measurements were performed in the Bone Disease Center at the Lausanne University Hospital (CHUV), Lausanne, Switzerland (TBS iNsight® Software version 1.8, Med-Imaps, Pessac, France) using anonymized spine DXA files to ensure blinding of the Swiss investigators to all clinical parameters and outcomes. The approach was similar to the one used in other studies [7]. BMD and TBS were evaluated at baseline and at follow-up. FRAX® score was calculated utilizing the clinical information from patients’ charts, and using the online algorithm [9].

BMD was interpreted using World Health Organization (WHO) guidelines, which define risk according to T-score, which is the standard deviation difference between a patient's BMD and that of a young-adult reference population. A T-score of ≤−2.5 indicates clinical osteoporosis. Osteopenia is defined as a borderline T-score (between −1.0 and −2.5), whereas normal BMD is defined as T-score >−1.0.

TBS, being a continuous variable as BMD, was interpreted using the tertile approach extracted from the fracture data of a large Canadian cohort. Degraded microarchitecture represents the highest risk, and is defined as a TBS value of ≤1.2. Partially degraded microarchitecture represents borderline risk, and is defined as values between 1.2 and 1.35, whereas normal microarchitecture is defined as TBS ≥1.35 [6], [7].

FRAX® assessment was conducted via retrospective medical records review and calculated through the online algorithm (https://www.shef.ac.uk/FRAX).

### 2.3 Definition of at-risk populations

Using BMD, TBS, and FRAX®, we evaluated three screening paradigms for identifying patients with high fracture risk who would be suitable for pharmacologic therapy with a BMA. The first screening paradigm is BMD alone using osteoporosis (T≤−2.5) as a threshold for positivity. The National Osteoporosis Foundation recommends BMA therapy for this population based upon models that predict a favorable cost-benefit ratio [10].

The second screening paradigm is BMD plus FRAX®, which is the standard screening practice endorsed by the National Comprehensive Cancer Network (NCCN) and National Osteoporosis Foundation [10]. For BMD plus FRAX®, the threshold for positivity is either osteoporosis by BMD, or osteopenia by BMD plus a FRAX 10-year probability of a hip fracture ≥3% or a 10-year probability of a major osteoporosis-related fracture ≥20%. These thresholds were determined based upon modeling predicting favorable cost-benefit ratio, specific to the United States population [10].

Finally, we tested a novel screening paradigm of BMD plus FRAX® plus TBS. For this method, we defined positivity to elevated fracture risk (EFR) as either: 1) osteoporosis by BMD (T-score ≤−2.5); 2) osteopenia + high FRAX® score (as above); or 3) osteopenia + low TBS score (degraded microarchitecture, i.e. TBS ≤1.2).

### 2.4 Statistical analysis

Statistical analysis was performed using IBM Statistical Package for the Social Sciences (SPSS)®. 0.05 was set as a threshold for statistical significance. Kolmogorov-Smirnov test ascertained normal distribution of data. Pearson's correlation was calculated to assess the association between TBS and BMD, one-way repeated measure ANOVA (using Mauchly's test to assess assumption of sphericity) and simple linear regression to assess prediction of TBS total percentage change by TBS at baseline, BMI, race, age at beginning of treatment, DXA interval and type of AI. All comparisons were two-tailed.

## 3 Results

### 3.1 Subject characteristics

Baseline demographic and clinical characteristics, as well as TBS and lumbar spine (LS) BMD values, are summarized in Table 1. Median age was 67 years (range 51–87 years) and median BMI was 27.5 kg/m^2^ (range 18.8–36.6 kg/m^2^). The majority of patients were treated with a non-steroidal AI (95%: 70, anastrozole; 24, letrozole; 1, letrozole followed by anastrozole), whereas 5% of patients were treated with a steroidal AI (5, exemestane).

**Table 1:** Trends in BMD and TBS, organized by clinical and demographic characteristics.

Group	LS BMD, Baseline	TBS, Baseline	TH BMD, baseline	ΔLS BMD/yr	ΔTBS/yr
All Patients	−0.04	1.29	−0.39	−0.45 (−1.5%)	−0.15 (−0.5%)

Age					
≤65 y (n=39)	−0.08	1.32	−0.17	−0.12 (−1.2%)	−0.01 (−0.8%)
>65 (n=61)	−0.01	1.27	−0.52	−0.14 (−1.7%)	−0.01 (−0.3%)

BMI					
BMI≤25 (n=30)	−0.51	1.3	−0.73	−0.16 (−1.3%)	−0.01 (−0.6%)
BMI>25 (n=70)	0.16*	1.28	−0.24	−0.12 (−1.5%)	−0.01 (−0.4%)

Ethnicity					
Caucasian (n=51)	−0.23	1.32	−0.7	−0.15 (−2.3%)	−0.01 (−0.9%)
Other (n=49)	0–17	1.26	−0.05	−0.12 (−1.9%)	0.00 (0%)

AI type					
Anastrozole (n=70)	−0.06	1.29	−0.44	−0.12 (−1.3%)	−0.01 (−0.8%)
Exemestane (n=5)	−0.24	1.32	0.22	−0.24 (−2.8%)	−0.02 (−1.4%)
Letrozole (n=24)	0.34	1.27	−0.39	−0.17 (−1.7%)	0.01 (0.9%)

Baseline LS BMD					
T≤−1.0 (n=35)	−1.71	1.26	−1.03	−0.09 (1.8%)	−0.01 (−0.2%)
T>−1.0 (=65)	0.86*	1.3	−0.05	−0.16 (1.3%)	−0.01 (−0.6%)

Baseline TBS					
TBS≤1.35 (n=68)	−0.24	1.22	−0.49	−0.14 (−1.3%)	0.02 (0.4%)
TBS>1.35 (n=32)	0.39	1.43*	−0.18	−0.11 (−1.8%)	0.03 (−2.2%)*

Legend: LS, lumbar spine; BMD, bone mineral density; TBS, trabecular bone score; ∆, change; y, year; AI, aromatase inhibitor.

* p<0.05/

At baseline, 45 subjects had normal BMD (45%), 51 subjects met WHO criteria for osteopenia (51%), and 4 subjects met criteria for osteoporosis (4%). By TBS, 33 subjects had normal bone microarchitecture (33%), whereas 47 subjects had partially degraded microarchitecture (47%) and 20 subjects had degraded microarchitecture (20%).

### 3.2 Analysis of combined screening approach of BMD+FRAX+TBS at baseline

We assessed the ability of the three screening approaches (BMD alone, BMD + FRAX®, or combined BMD + FRAX® + TBS) to identify post-menopausal breast cancer patients at risk of fragility fracture. At baseline DXA, only 4% of patients (n=4/100) met criteria for EFR by BMD alone corresponding to a number needed to screen (NNS=#screened /#diagnosed) of 25. With the addition of the FRAX® assessment tool, 13% of patients (n=13/100) met criteria for EFR, corresponding to a NNS of 8. Finally, with the combined approach including TBS, 20% of patients (n=20/100) met criteria for EFR, corresponding to a NNS of 5. Fig. 2a illustrates the breakdown of how subjects are classified according to each of the screening approaches.

### 3.3 Analysis of combined screening approach of BMD+FRAX®+TBS during AI therapy

We then evaluated the ability of each of the screening approaches to detect AI-associated fracture risk among patients who did not have EFR at baseline. In this data series, follow-up DXA was performed on average 21 months following initiation of AI (12–35 months). Eighty subjects did not exhibit EFR at baseline. At follow-up, one additional subject developed EFR by BMD criteria, versus an additional 3 subjects by BMD + FRAX® criteria, versus an additional 7 subjects by BMD + FRAX® + TBS criteria. Despite less profound percentage changes in TBS on average across all patients, changes in TBS were more likely to influence the determination of EFR following initiation of AI. Fig. 2b illustrates the breakdown according to each of the screening approaches. In summation, a BMD-only screening approach with baseline and follow-up DXA exhibited a NNS of 20, versus NNS of 6 for BMD plus FRAX®, versus NNS of 4 for BMD plus FRAX ®plus TBS.

### 3.4 Effect of AI on TBS and BMD

AI therapy was associated with statistically significant declines in lumbar spine BMD (mean annual decline: −0.45 T-score units, or −1.5%; p<0.001) but not in TBS (mean annual decline: −0.15 units, or −0.5%, NS).

Neither age, ethnicity, BMI, nor AI type appeared to influence baseline BMD, TBS, or changes in BMD or TBS following AI (Table 1). However, the TBS score at baseline influenced the likelihood of TBS declines associated with AI therapy: subjects with low TBS scores at baseline (as defined by degraded microarchitecture, i.e. TBS ≤1.2) were less likely to experience further declines in TBS, whereas subjects with normal TBS at baseline were more likely to experience declines in TBS. This finding reached statistical significance (p<0.1), with mean percentage declines of normal TBS group being −3.7%, versus −0.9% in the partially degraded microarchitecture group and +3.9% in the degraded microarchitecture group. Conversely, declines in BMD did not appear to be influenced by baseline BMD.

### 3.5 BMD and TBS are differentially influenced by AI therapy

**Fig. 1:**
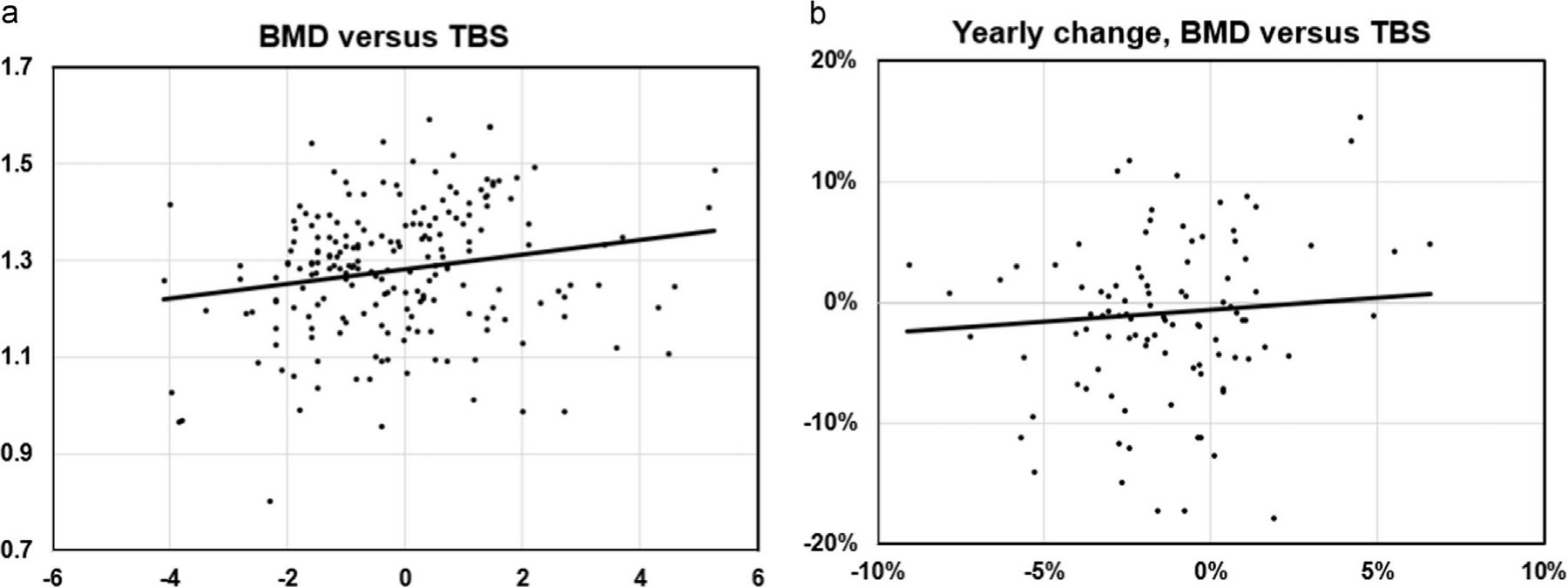
Correlations of BMD with TBS. **A:** Correlation of intra-patient BMD (lumbar spine) versus TBS at baseline; **B:** Correlation of changes in BMD versus TBS over time, calculated as annual rate of change. Legend: BMD, bone mineral density; TBS, trabecular bone score.

We then evaluated whether BMD and TBS were independent variables in this dataset. Fig. 1 illustrates the correlation between TBS values and lumbar spine BMD, with each data point representing an individual DXA scan. We observed a weak positive relationship between BMD and TBS (Pearson r=0.19, p=0.007), indicating that subjects with preserved BMD may be more likely to have non-degraded bone microarchitecture. We next evaluated for correlations in changes in BMD versus TBS following initiation of AI. To control for variations in the time interval between serial DXA scans, we plotted the annual percentage change in BMD versus the annual percentage change in TBS. With this approach, we found no correlation in change in BMD versus change in TBS (Pearson r=0.07, NS), suggesting that AI may differentially influence BMD versus TBS within individual patients. Furthermore, no correlations were identified when repeated for absolute annual change (i.e. change in BMD/yr versus change in TBS/yr), or non-adjusted changes (i.e. change in BMD versus change in TBS, or % change in BMD versus % change in TBS) (data not shown).

### 3.6 The majority of cases of EFR are detected when follow-up DXAs are performed greater than 18 months following initiation of AI

Substantial heterogeneity exists in clinical practice with regards to the timing of repeat DXA following the initiation of AI. In our dataset, we wished to ascertain and compare the rate of detection of acquired EFR with an early detection strategy (i.e. DXA within 18 months of AI initiation) versus a delayed detection strategy (i.e. DXA >18 months following AI initiation). Excluding patients who had high fracture risk at baseline, 30% of subjects (n=24/80) were evaluated with an “early” DXA (interval range 11–18 months, average 13.8, SD 2.2), whereas 70% of subjects (n=56/80) were evaluated with a “delayed” DXA (interval range 18–35 months, average 24.5, SD 3.2). In the early DXA group, only one subject (4%) developed EFR following AI (as identified by a decrease in TBS). Contrastingly, the majority of EFR was detected in the delayed DXA group, with 6 patients detected (11%) ( Fig. 3). These data correspond with a NNS of 24 associated with early DXA scans, versus 13 for delayed DXA scans.

**Fig. 2:**
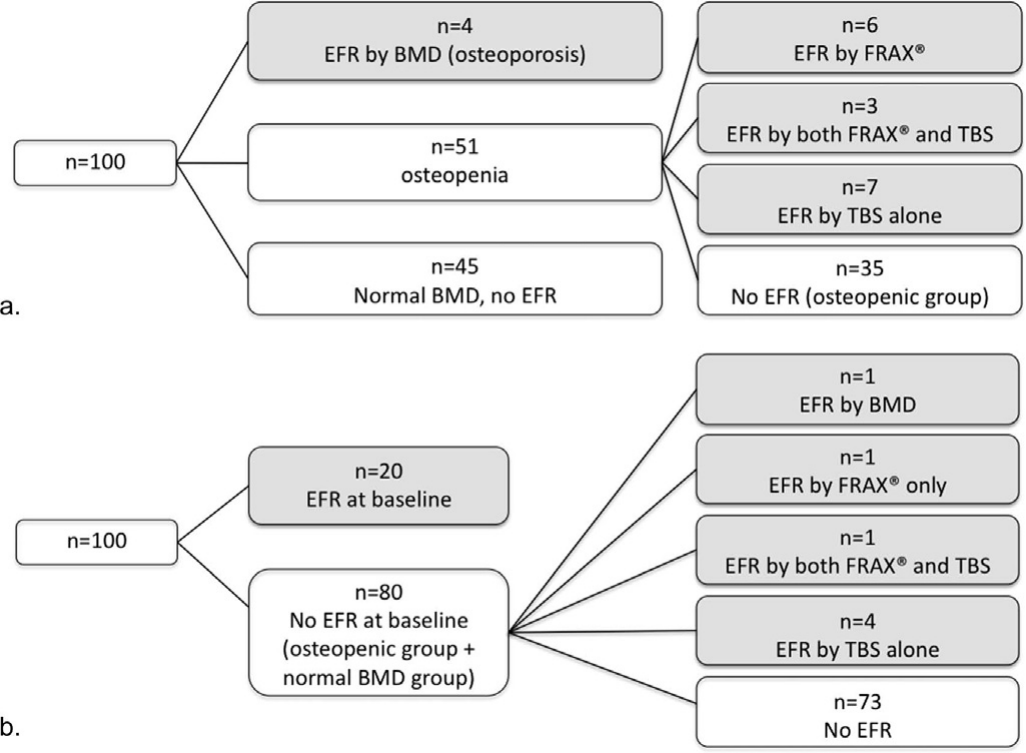
Detection of EFR using various screening strategies. **A:** Distribution of EFR patients detected on baseline DXA, by BMD alone, BMD+FRAX®, or BMD+FRAX®+TBS; **B:** Distribution of EFR detected on follow-up DXA. Legend: DXA, dual-energy X-ray absorbimetry; BMD, bone mineral density; FRAX®, fracture risk assessment tool; TBS, trabecular bone score. Gray boxes indicate designation of EFR.

**Fig. 3:**
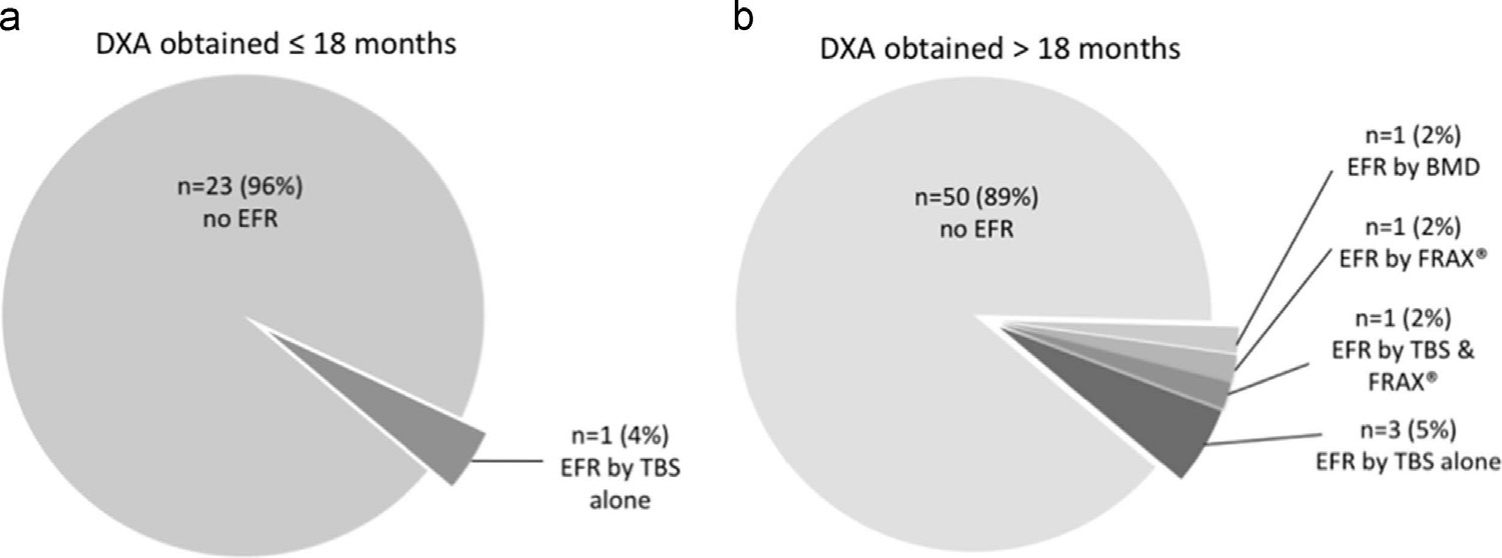
Detection rates of AIs -associated fracture risk, by timing of follow up DXA. **A:** Detection rate in patients evaluated ≤18 months from initiation of AI; **B:** Detection rate in patients evaluated >18 months from initiation of AI. Legend: DXA, dual-energy X-ray absorbimetry; AI, aromatase inhibition; BMD, bone mineral density; FRAX, fracture risk assessment tool; TBS, trabecular bone score.

## 4 Discussion

Patients treated with AIs are at increased risk of fragility fractures, and BMD assessment alone is unable to identify all patients at risk. Recently, numerous alternative risk-assessment tools have been developed to better estimate fracture risk, which may allow for a more efficient preventative approach in patients receiving AI.

The most extensively validated tool is the FRAX®, a computer-based algorithm that calculates risk of fracture based on BMD and clinical factors [11]. In the setting of AI therapy, FRAX® facilitates the decision of whether to administer BMAs in women with borderline BMD (osteopenia). A major advantage of FRAX® is that it utilizes readily-available clinical information. FRAX®, however does not specifically assess the risk of fracture in women with breast cancer treated with AIs and may underestimate their effects on bone [12].

Furthermore, methodologies that assess bone quality and predict fracture risk have emerged. A large body of evidence validates the utility of TBS in predicting fragility fracture [13], [14]. In the largest longitudinal series, the Manitoba study, TBS and BMD were evaluated in 29,407 women (>50 years of age) and followed for fragility fractures. With a median follow up of 4.7 years, TBS emerged as a highly significant predictor of fracture. Women with osteopenia and low TBS values exhibited fracture rates on par with that of women with osteoporosis, suggesting that TBS may also help facilitate the decision to treat with a BMA [7]. Such findings were corroborated in an international meta-analysis of 14 studies (excluding the Manitoba cohort) — together incorporating 17,809 men and women (59% women) ranging in age from 40 to 90 years (mean age 72). The gradient of risk per standard deviation decrease of TBS for hip fracture or other major osteoporotic fracture (clinical spine, distal forearm or proximal humerus fracture) ranged from 1.31 to 1.54 depending on age and fracture outcome with no difference between men and women. Such level of risk remained significant even after adjustment for both spine BMD and clinical risk factors [8].

We conducted a retrospective analysis to compare a BMD-alone strategy, versus BMD+FRAX® and BMD+FRAX®+TBS screening strategies. To our knowledge, this constitutes the first study to assess the relative contributions of FRAX® and TBS. In our cohort, FRAX®+BMD identified 9 (9%) additional patients at increased risk for fractures at baseline and 2 (2%) after AI treatment compared to BMD alone, thus corroborating the current National Comprehensive Cancer Network and National Osteoporosis Foundation guidelines to conduct FRAX® assessment in osteopenic women who require AI [10]. Likewise, the addition of TBS increased the numbers of at-risk patients detected both at baseline (an additional 16, 16%) and at follow-up (an additional 6, 6%) compared to BMD alone.

Consistent with other reports [15], [17], the effect of AI on TBS did not appear to correlate with changes in BMD, suggesting that these two risk assessments may be complementary rather than overlapping, and could potentially be combined to create a more sensitive measure of bone fragility. Our conclusions are corroborated by two small previous studies evaluating the influence of AI on TBS. In one study, 34 breast cancer patients treated with AI were evaluated with both BMD and TBS, and sustained a decrease of 5.9% and 2.1%, respectively, across a mean DXA interval of 2.1 years (corresponding to a rate of loss of 2.8% and 1% annually) [15]. Another study evaluated the effect of exemestane on TBS in 19 patients, and showed a TBS decrease of 2.3% at 24 months (corresponding to a mean annual decrease of 1.2%) [16]. These findings are generally consistent with our study, which demonstrated mean annual decreases of 1.5% for BMD and 0.5% for TBS.

One unique finding from our dataset is that subjects with normal TBS at baseline are more likely to experience declines in TBS, whereas subjects with low TBS at baseline do not experience further declines. We speculate that this finding may reflect variations in sensitivity of the measurement assay across high versus low TBS ranges, and indicate that the TBS measurement platform may have highest sensitivity for detecting changes in the normal range. This finding, combined with the finding that TBS contributed the most to identifying patients’ AI-associated fracture risk, supports a practice of obtaining serial TBS measurements in patients with normal TBS at baseline, but may argue that serial measurement in patients with low TBS is less informative, as this population might already be at high risk of fracture.

In our study, the decline in TBS during AIs treatment was not statistically significant. Changes in TBS after osteoporosis therapy are known to be of lesser magnitude than corresponding changes in BMD; conversely, it is possible that BMD losses in the setting of AI therapy occur in similar fashion [13]. Additionally, longer follow up might have demonstrated the decline in TBS associated with relatively longer use of AIs (mean 2.1 years) that others have demonstrated [15]. Furthermore, patients in our study with follow up DXA after >18 months of AI treatment were more likely to experience EFR than <18 months of follow up. Thus, serial TBS measurements appear to identify subjects with newly-acquired fracture risk following initiation of AI therapy with greater sensitivity following 18 months on AI. Future studies assessing TBS after longer follow up are needed to further evaluate AI-associated changes in trabecular microstructure over five to ten years.

In summary, our analysis supports further evaluation of a screening approach of BMD+FRAX®+TBS, obtained at baseline and at 2 years following AI, with special attention paid to patients with osteopenia and non-degraded bone microarchitecture by TBS. An alternative approach could be the use of TBS-adjusted FRAX BMD as it has been recently proposed [8]. This later approach would still have to be tested in this very specific population.

Recently published European consensus guidelines suggest that the use of bisphosphonates should be considered for the prevention of cancer treatment-induced bone loss in all patients with a T-score of <−2.0 [17]. According to this approach, 7 additional patients would have been identified at baseline in our population based on DXA alone, of these 2 would have been identified by the combined approach of osteopenia + low TBS score, 2 by osteopenia + high FRAX® score, and 1 by both osteopenia + low TBS score and osteopenia + high FRAX®. Only 28% of patients identified by the combined approach osteopenia + low TBS score would have been identified by lowering the T-score threshold. This result suggests that modification of T-score threshold would not hamper the usefulness of TBS.

Our findings should be interpreted in the context of recently reported phase III studies, evaluating the potential utility of a universal BMA treatment approach for all post-menopausal women receiving AI. Recent large randomized studies in adjuvant AI treated breast cancer patients have demonstrated, respectively: an improvement in BMD with risedronate, as well as both an improvement in BMD plus a reduction in fragility fractures with denosumab [18]. (Gnant et al., n.d.) These findings, while clearly demonstrating the clinical utility of BMAs, do not obviate the importance of finding better strategies to identify patients at risk of fragility fractures. An approach of universally treating all patients starting therapy with an AI with a BMA has not been adopted. Indeed, the majority of women will not experience a fragility fracture, and therefore a universal treatment approach would lead to overtreatment with BMAs, which may be costly and associated with toxicities. When considering extended treatment with BMAs, rare adverse effects such as atypical femur fractures and osteonecrosis of the jaw, likely related to duration of use, should be taken in consideration [19]. An effective screening approach may serve to better identify patients who may benefit from BMAs.

Our study is limited by the number of subjects and its retrospective nature. Additionally, an optimal TBS threshold for determining risk has not been firmly established. The TBS categories used in our study have been recently proposed by an international working group of TBS users, and correspond to the tertiles for fracture risk as defined in a recent meta-analysis by McCloskey et al. In this study the thresholds of 1.23 and 1.31 defined high risk and intermediate risk respectively, as the risk for major osteoporotic fracture were significantly higher in the highest and intermediate tertile compared with the lowest-risk tertile (TBS >1.31) [[8], [20]]. (“MEDIMAPS-UK-WEB.pdf,” n.d).

## 5 Conclusion

In conclusion, our retrospective analysis demonstrates that TBS combined with the standard-of-care risk assessment tools BMD + FRAX® may identify additional patients at high risk of fragility fracture both before and during treatment with AIs, who may benefit of treatment with BMAs.

## Competing interests

DH is co-owner of TBS patent and has corresponding ownership shares and position in medimaps group. All the other authors declare that they have no competing interests.

## Authors’ contributions

VM and DP are co-first authors of this work, they contributed equally.

MF and AF are co-senior authors of this work.

## References

[1] Coates A.S., Keshaviah A., Thürlimann B., Mouridsen H., Mauriac L., Forbes J.F., Paridaens R., Castiglione-Gertsch M., Gelber R.D., Colleoni M., Láng I., Mastro L.D., Smith I., Chirgwin J., Nogaret J.-M., Pienkowski T., Wardley A., Jakobsen E.H., Price K.N., Goldhirsch A. (2007). Five Years of Letrozole Compared With Tamoxifen As Initial Adjuvant Therapy for Postmenopausal Women With Endocrine-Responsive Early Breast Cancer: Update of Study BIG 1-98. J. Clin. Oncol..

[2] Adjuvant bisphosphonate treatment in early breast cancer: meta-analyses of individual patient data from randomised trials Early Breast Cancer Trialists’ Collaborative Group (EBCTCG)*Lancet 2015; 386: 1353–61 10.1016/S0140-6736(15)60908-4 26211824

[3] Gnant, M., Pfeiler, G., Dubsky, P.C., Hubalek, M., Greil, R., Jakesz, R., Wette, V., Balic, M., Haslbauer, F., Melbinger, E., Bjelic-Radisic, V., Artner-Matuschek, S., Fitzal, F., Marth, C., Sevelda, P., Mlineritsch, B., Steger, G.G., Manfreda, D., Exner, R., Egle, D., Bergh, J., Kainberger, F., Talbot, S., Warner, D., Fesl, C., Singer, C.F., n.d. Adjuvant denosumab in breast cancer (ABCSG-18): a multicentre, randomised, double-blind, placebo-controlled trial. The Lancet. doi:10.1016/S0140-6736(15)60995-3

[4] Schuit S.C.E., van der Klift M., Weel A.E.A.M., de Laet C.E.D.H., Burger H., Seeman E., Hofman A., Uitterlinden A.G., van Leeuwen J.P.T.M., Pols H.A.P. (2004). Fracture incidence and association with bone mineral density in elderly men and women: the Rotterdam Study. Bone.

[5] Szulc P., Delmas P.D. (2008). Biochemical markers of bone turnover: potential use in the investigation and management of postmenopausal osteoporosis. Osteoporos. Int. J. Establ. Result Coop. Eur. Found. Osteoporos. Natl. Osteoporos. Found. USA.

[6] Silva B.C., Leslie W.D., Resch H., Lamy O., Lesnyak O., Binkley N., McCloskey E.V., Kanis J.A., Bilezikian J.P. (2014). Trabecular bone score: a noninvasive analytical method based upon the DXA image. J. Bone Miner. Res. Off. J. Am. Soc. Bone Miner. Res..

[7] Hans D., Goertzen A.L., Krieg M.-A., Leslie W.D. (2011). Bone microarchitecture assessed by TBS predicts osteoporotic fractures independent of bone density: the Manitoba study. J. Bone Miner. Res. Off. J. Am. Soc. Bone Miner. Res..

[8] McCloskey E.V., Odén A., Harvey N.C., Leslie W.D., Hans D., Johansson H., Barkmann R., Boutroy S., Brown J., Chapurlat R., Elders P.J., Fujita Y., Glüer C.-C., Goltzman D., Iki M., Karlsson M., Kindmark A., Kotowicz M., Kurumatani N., Kwok T., Lamy O., Leung J., Lippuner K., Ljunggren Ö., Lorentzon M., Mellström D., Merlijn T., Oei L., Ohlsson C., Pasco J.A., Rivadeneira F., Rosengren B., Sornay-Rendu E., Szulc P., Tamaki J., Kanis J.A. (2015). A meta-analysis of trabecular bone score in fracture risk prediction and its relationship to FRAX. J. Bone Miner. Res. Off. J. Am. Soc. Bone Miner. Res..

[9] Task Force of the FRAX Initiative, Kanis J.A., Hans D., Cooper C., Baim S., Bilezikian J.P., Binkley N., Cauley J.A., Compston J.E., Dawson-Hughes B., El-Hajj Fuleihan G., Johansson H., Leslie W.D., Lewiecki E.M., Luckey M., Oden A., Papapoulos S.E., Poiana C., Rizzoli R., Wahl D.A., McCloskey E.V. (2011). Interpretation and use of FRAX in clinical practice. Osteoporos. Int.

[10] Cosman F., de Beur S.J., LeBoff M.S., Lewiecki E.M., Tanner B., Randall S., Lindsay R. (2014). Clinician’s Guide to Prevention and Treatment of Osteoporosis. Osteoporos. Int. J. Establ. Result Coop. Eur. Found. Osteoporos. Natl. Osteoporos. Found. USA.

[11] Silverman S.L., Calderon A.D. (2010). The utility and limitations of FRAX: A US perspective. Curr. Osteoporos. Rep.

[12] Paterson A.H.G. (2006). The role of bisphosphonates in early breast cancer. The Oncologist.

[13] Silva B.C., Broy S.B., Boutroy S., Schousboe J.T., Shepherd J.A., Leslie W.D. (2015). Fracture Risk Prediction by Non-BMD DXA Measures: the 2015 ISCD Official Positions Part 2: Trabecular Bone Score. J. Clin. Densitom. Off. J. Int. Soc. Clin. Densitom..

[14] Harvey N.C., Glüer C.C., Binkley N., McCloskey E.V., Brandi M.-L., Cooper C., Kendler D., Lamy O., Laslop A., Camargos B.M., Reginster J.-Y., Rizzoli R., Kanis J.A. (2015). Trabecular bone score (TBS) as a new complementary approach for osteoporosis evaluation in clinical practice. Bone.

[15] Pedrazzoni M., Casola A., Verzicco I., Abbate B., Vescovini R., Sansoni P. (2014). Longitudinal changes of trabecular bone score after estrogen deprivation: effect of menopause and aromatase inhibition. J. Endocrinol. Invest..

[16] Kalder M., Hans D., Kyvernitakis I., Lamy O., Bauer M., Hadji P. (2014). Effects of Exemestane and Tamoxifen treatment on bone texture analysis assessed by TBS in comparison with bone mineral density assessed by DXA in women with breast cancer. J. Clin. Densitom. Off. J. Int. Soc. Clin. Densitom..

[17] Hadji P., Coleman R.E., Wilson C., Powles T.J., Clézardin P., Aapro M., Costa L., Body J.-J., Markopoulos C., Santini D., Diel I., Leo A.D., Cameron D., Dodwell D., Smith I., Gnant M., Gray R., Harbeck N., Thurlimann B., Untch M., Cortes J., Martin M., Albert U.-S., Conte P.-F., Ejlertsen B., Bergh J., Kaufmann M., Holen I. (2016). Adjuvant bisphosphonates in early breast cancer: consensus guidance for clinical practice from a European Panel. Ann. Oncol.

[18] Cuzick J., Sestak I., Forbes J.F., Dowsett M., Knox J., Cawthorn S., Saunders C., Roche N., Mansel R.E., von Minckwitz G., Bonanni B., Palva T., Howell A., IBIS-II investigators (2014). Anastrozole for prevention of breast cancer in high-risk postmenopausal women (IBIS-II): an international, double-blind, randomised placebo-controlled trial. Lancet Lond. Engl.

[19] Bone H.G., Chapurlat R., Brandi M.-L., Brown J.P., Czerwinski E., Krieg M.-A., Mellström D., Radominski S.C., Reginster J.-Y., Resch H., Ivorra J.A.R., Roux C., Vittinghoff E., Daizadeh N.S., Wang A., Bradley M.N., Franchimont N., Geller M.L., Wagman R.B., Cummings S.R., Papapoulos S. (2013). The effect of three or six years of denosumab exposure in women with postmenopausal osteoporosis: results from the FREEDOM extension. J. Clin. Endocrinol. Metab..

[20] MEDIMAPS-UK-WEB.pdf [WWW Document], n.d. URL 〈http://www.medimapsgroup.com/upload/MEDIMAPS-UK-WEB.pdf〉 (accessed 9.29.15).

